# Relationship between nasopharyngeal and bronchoalveolar microbial communities in clinically healthy feedlot cattle

**DOI:** 10.1186/s12866-017-1042-2

**Published:** 2017-06-23

**Authors:** Mohamed M. Zeineldin, James F. Lowe, Elsbeth D. Grimmer, Maria R. C. de Godoy, Mohamed M. Ghanem, Yassein M. Abd El-Raof, Brian M. Aldridge

**Affiliations:** 1 Integrated Food Animal Management Systems, Department of Veterinary Clinical Medicine, College of Veterinary Medicine, University of Illinois at Urbana-Champaign, 241 LAC, 1008 W Hazelwood Dr, Urbana, IL 61802 USA; 2 Department of Animal Sciences and Division of Nutritional Sciences, University of Illinois at Urbana-Champaign, Urbana, USA; 3 Department of Animal Medicine, College of Veterinary Medicine, Benha University, Benha, Egypt

**Keywords:** Feedlot, Microbiota,16S rRNA gene, Next generation sequencing, Respiratory tract

## Abstract

**Background:**

The importance of upper airway structure in the susceptibility of the lower respiratory tract to colonization with potential pathogens is well established. With the advent of rapid, high throughput, next generation sequencing, there is a growing appreciation of the importance of commensal microbial populations in maintaining mucosal health, and a realization that bacteria colonize anatomical locations that were previously considered to be sterile. While upper respiratory tract microbial populations have been described, there are currently no published studies describing the normal microbial populations of the bovine lower respiratory tract. Consequently, we have little understanding of the relationship between upper and lower respiratory tract microbiota in healthy cattle. The primary objective of our study was to characterize the composition, structure and relationship of the lower and upper respiratory microbial communities in clinically healthy feedlot cattle. Nasopharyngeal swabs (NPS), and bronchoalveolar lavage (BAL) fluid, were collected from clinically healthy feedlot calves (*n* = 8). Genomic DNA from each sample was extracted, and the V3-V4 hypervariable region of the bacterial 16S rRNA gene was amplified and sequenced using Illumina Miseq platform.

**Results:**

Across all samples, the most predominant phyla were *Proteobacteria, Actinobacteria* and *Firmicutes.* The most common genera were *Rathayibacter, Mycoplasma, Bibersteinia* and *Corynebacterium.* The microbial community structure was distinct between these two biogeographical sites. Most of the bacterial genera identified in the BAL samples were also present in the NPS, but biogeographical-specific genera were enriched in both the NPS (*Rathayibacter*) and BAL (*Bibersteinia)* samples. There were strong associations between the presence of certain taxa at each specific location, and strong correlations between the presence of specific taxa in both the NPS and BAL samples.

**Conclusions:**

The correlation between the presence of specific taxa in both the NPS and BAL samples, supports the notion of a mutualistic interrelationship between these microbial communities. Future studies, in large cohorts of animals, are needed to determine the role and clinical importance of the relationships of respiratory tract microbial communities with health, productivity, and susceptibility to the development of respiratory disease, in growing cattle.

**Electronic supplementary material:**

The online version of this article (doi:10.1186/s12866-017-1042-2) contains supplementary material, which is available to authorized users.

## Background

Bovine respiratory disease (BRD) is one of the most commonly reported diseases in cattle, especially in intensely raised, recently weaned and newly transported feedlot cattle [1]. The etiopathogenesis of BRD is complex, and results from the complex interaction of bacterial and viral pathogens under the influence of a wide range of host and environmental risk factors [2]. Despite many advancements in management and therapeutics, BRD and its sequelae, continue to be the leading causes of animal morbidity, mortality, welfare concern and production loss to the industry [3]. The importance of the mucosal microbial community structure in maintaining epithelial health and homeostasis of the skin, gastrointestinal and reproductive tracts has been recognized for many years, but its significance has only relatively recently been demonstrated in the respiratory tract [4]. Despite the implication of resident bacterial populations in the etiopathogenesis of BRD [5], the detailed structure of the lower airway microbiota has not been investigated. The characterization of airway microbiota is expected to provide insights into the pathophysiology of BRD, and open the door for new health management strategies. Historically, characterization of the cattle microbiota has relied heavily on culture-dependent techniques which have mainly focused on the identification of major pathogens that can be easily cultured. While useful, this approach is unable to provide information on those organisms that cannot be easily cultured, but are likely present at these sites, and so only provides a narrow understanding of the complexity of these clinically important microbial ecosystems [6]. Advances in next generation sequencing and bioinformatics have facilitated the characterization of the composition and diversity of complex microbial populations at mucosal sites, and have provided remarkable insight into the interaction of these populations with the host [7]. Most next generation sequencing studies in cattle have focused on the gastrointestinal tract [8, 9], but the existence of important resident microbial populations in the nasopharynx has also been described [10]. The microbiota of the respiratory tract is of particular interest due to its association with BRD [5]. It is widely recognized that the upper respiratory tract microbiome provides a first line of defense against foreign invaders through competition, and interaction, with potential mucosal pathogens [11]. While the composition and development of the nasopharyngeal microbiota in feedlot cattle has been recently described [5, 10, 12, 13], there are no published studies describing lower respiratory tract microbial communities in feedlot cattle. Interestingly, until recently the lower respiratory tract in healthy humans has traditionally been considered as sterile using culture-dependent or conventional molecular techniques [14, 15]. Technical advances in culture-independent techniques have shown that the respiratory tract of healthy people is not sterile, but is composed of a complex and previously unappreciated microbial community [16, 17], and that certain configurations of the microbiota may be associated with development of respiratory disease [18]. In view of the importance of respiratory disease to the industry, our objectives were to characterize the composition and structure of the upper and lower respiratory microbiota, and to determine the relationship between the nasopharyngeal and bronchoalveolar microbiotas in clinically healthy feedlot cattle.

## Methods

### Animal populations and sample collection

A total of eight, six to eight month old, single-source, Charolais feedlot calves (mean body weight 348.47 ± 14.59 kg), were enrolled in our study in April 2016, approximately 60 days after arrival at the South Farms-Beef Cattle and Sheep Field Laboratory, University of Illinois at Urbana- Champaign- USA. All calves were clinically healthy, with no history of receiving any antimicrobial drugs prior to, or after arrival at the feedlot. The calves were vaccinated immediately after arrival at the feedlot, with a modified live virus vaccine against IBR, BVDV (Types I and II), BPIV-3 and BRSV (5 ml IM; Bovi-Shield Gold FP5 L5 HB Cattle Vaccine, Zoetis Animal Health), and dewormed with a topical anthelminthic (Noromectin Pour-On Solution, Norbrook® Inc. USA). The calves were fed a mixed ration (20% silage, 20% modified wet distillers grains with soluble, 10% dry supplement, and 50% high moisture corn), and given free access to water. The use of animals for this study was approved by the University of Illinois Institutional Animal Care and Use Committee (IACUC Protocol: #15064) and all of the experimental protocols were performed in accordance with relevant guidelines and regulations set by IACUC.

Physical exams, including rectal temperature, respiratory rate and pulse rate, were performed with the calves standing in the hydraulic chute. A clinical lung score was recorded using the microphone of an automated Whisper stethoscope (Whisper®, Geissler Corp, Plymouth, MN). Briefly, the stethoscope was placed over the 5th intercostal space of the right thoracic wall, approximately 10 cm above the elbow, at a site known to encompass the apical lung lobes. Recorded lung sounds were then automatically transmitted wirelessly to a computer located within 2 m of the stethoscope, and analyzed using software rendering a 5-point lung score scales [19]. The program software is designed to remove heart sounds and potential interference from the environment, and classifies acoustic patterns in to lung scores ranging from 1 to 5 (1 = normal, 2 = mild acute, 3 = moderate acute, 4 = severe acute, and 5 = chronic).

Feedlot calves with a rectal temperature < 39.4 °C, respiratory rate < 50 breaths/min, pulse rate < 120 beats/min and lung score ≤ 2 were defined as healthy, and selected for sampling.

Following physical examination, a single, deep NPS was collected from each calf, using a 33-in.-long double guarded PVC culture swab (Kalayjian Industries, Inc. U.S.A.) according to published techniques [20]. Briefly, the calf’s nostrils were cleaned with a disposable wipe before collection. The NPS was carefully inserted into the ventral meatus of the nose, and advanced approximately 2/3 of the dorsal head length. Once in place, the end of the swab was exposed to a length of approximately 1–2 in., by withdrawing the outer sleeve, and the sampling unit was then firmly rotated through 360^°^ against the pharyngeal wall, for 20–30 s. The cotton tipped swab was then retracted back in to the outer sheath, and the whole swab was removed gently from the animal’s nose. Each cotton swab was then broken off into a sterile 2 ml cryo-tube, transported on dry ice to the laboratory, and stored at −20 °C pending further processing.

Following NPS sampling, the calves were sedated with Xylazine hydrochloride (0.1 mg/kg IM) (Rompun®, Bayer health care LLC, Kansas) and the nostrils were cleaned with dry gauze. BAL was performed with the sedated calves in a standing position, using a sterilized, flexible BAL tube (BAL300 – 300 cm length, 10 mm outside diameter, 2.5 mm internal diameter, balloon volume 10 cm^3^, MILA International, Inc. U.S.A.) [21]. The BAL catheter was introduced into the nostril, directed into the ventral meatus, and then advanced until it encountered resistance in the caudal pharynx. At this point, the calf’s head and neck were held straight, in maximal extension, to allow the catheter to pass into the trachea during the inspiratory phase of the respiratory cycle. On reaching a wedged position in the lower respiratory airway, the catheter was secured by inflating the balloon cuff with 5 cm^3^ of air. For sampling, 120 mls of sterile saline was infused through the catheter lumen, using 60 ml syringes attached to a stopcock and catheter tipped adapter. Immediately after the 120 ml infusion, negative pressure was applied to aspirate airway fluid, which was immediately placed into a sterile 50 ml specimen tube. The BAL samples were stored on ice until processing, approximately 2–4 h after collection. The collected BAL was centrifuged at 14,000×g for 10 min. The pellets were re-suspended in 500 μL sterile PBS and stored at −20 °C pending further analysis.

### Genomic DNA extraction

Genomic DNA extraction was performed from each NPS and BAL sample using the PowerFecal® DNA isolation Kit (MO BIO Laboratories, Inc., Carlsbad, CA, USA), according to the manufacturer’s instructions. Briefly, each sample was transferred to a dry bead tube with 60 μl of Solution C1 and 750 μl of Bead Solution, heated at 65 °C for 10 min, and settled in Bullet Blender 24 Gold tube holder (Next Advance, Inc., Averill Park, NY, USA). The tubes were vortexed at maximum speed for 10 min to achieve microbial cell disruption. The PowerFecal® DNA isolation Kit protocol was used to complete the extraction, according to manufacturer instructions. Purified DNA was eluted into 50 μl of Solution C6 rather than 100 μl to increase the DNA concentration. Total DNA concentration was quantified using a Nanodrop™ spectrophotometer (NanoDrop Technologies, Rockland, DE, USA) at wavelengths of 260 and 280 nm, and the integrity was confirmed by agarose gel electrophoresis.

### Fluidigm access Array amplification of the V3-V4 hypervariable region of 16S rRNA genes and Illumina sequencing

Genomic DNA was subject to Fluidigm Access Array Amplification (Fluidigm Corporation, South San Francisco, CA, USA). Prior to amplification, all DNA samples were measured on a Qubit™ fluorometer (Life technologies, Grand Island, NY, USA) using the High Sensitivity DNA Kit (Agilent Technologies, Santa Clara, CA, USA) [22].

Briefly, the primer sequences F357-for (CCTACGGGAGGCAGCAG) and R806-rev (GGACTACNVGGGTWTCTAAT) were used to amplify the V3-V4 hypervariable region of the 16 s rRNA gene. PCR reactions were performed on a Fluidigm Biomark HD™ PCR machine (Fluidigm Corporation, South San Francisco, CA, USA) using the default Access Array cycling program without imaging.

Harvested product was quantified on a Qubit™ fluorometer (Life technologies, Grand Island, NY, USA) and stored at −20 °C. All samples were run on a Fragment Analyzer™ (Advanced Analytics, Ames, IA, USA), and the amplicon regions were quantified. PCR products were then size selected on a 2% agarose E-gel™ (Life technologies, Grand Island, NY, USA) and extracted from the isolated gel slice with the QIAquick Gel extraction kit (Qiagen, Valencia, CA, USA). Cleaned, size-selected product was examined on an Agilent Bioanalyzer™ to confirm appropriate profile, and for the determination of average size. The final pooled Fluidigm libraries were transferred to the DNA Services lab at the W. M. Keck Center for Comparative and Functional Genomics (University of Illinois at Urbana-Champaign, Urbana, IL, USA) for Illumina sequencing. The Illumina MiSeq™ platform (Illumina, San Diego, CA, USA) was used to sequence the V3- V4 region of the 16S rRNA gene according to the Illumina instructions.

### Sequence data processing and statistical analysis

The raw sequence data were preprocessed and analyzed using the open-source software package, Quantitative Insights Into Microbial Ecology (QIIME®) version 1.9 (http://qiime.org/) [23]. Sequences were filtered for quality the default parameters of the split_libraries.py command; minimum sequence length equal 200, maximum sequence length equal 1000, a Phred score of less than 25, maximum number of ambiguous bases equal 6 and homopolymer runs of >6 bp [24]. Chimeric sequences were detected and removed using UCHIME [25]. The remaining sequences were clustered into operational taxonomic units (OTUs) using open reference OTU selection protocols (97% identity cutoff) with the UCLUST algorithm [26], and assigned a taxonomic classification against the Greengenes® database [27].

The core microbiota, those microbes shared among all sampled calves, was identified at the genus level. For subsequent alpha and beta diversity analysis, the OTU table was randomly subsampled and rarefied to 2400 sequences per sample. Alpha diversity (an estimate of bacterial community richness in a sample) was calculated within QIIME® using the Chao1 (an estimate of species richness), observed species (the total number of microbial species present in a community), and phylogenetic diversity (PD whole tree) (an estimate of the biodiversity which incorporates phylogenetic difference between species). Beta-diversity (an estimate of bacterial communities expression of diversity between different sites) was calculated using weighted and unweighted UniFrac distance [28] and clustering of the samples based on distance was visualized on principal coordinate analysis (PCoA) plots using EMPeror® [29].

The OTU relative abundance values were analyzed using the linear discriminant analysis (LDA) effect size (LEfSe) algorithm, to identify OTUs that display significant differences between the two sites [30]. Additionally, a cladogram was produced using the online LEfSe tool. The algorithm first used the non-parametric factorial Kruskal-Wallis test to detect taxa with significantly different abundance, followed by pairwise Wilcoxon test to detect biological consistency between NPS and BAL samples, and then used LDA to estimate the effect size of each differentially abundant feature. Statistical analyses were performed using JMP® 12.12 (SAS Institute Inc., North Carolina, USA).

For characterization of NPS versus BAL microbiota, analysis of bacterial abundance between the NPS and BAL samples in healthy calves was performed using nonparametric Wilcoxon tests. Alpha diversity metrics (Chao1, observed species and PD whole tree) were also compared between the two groups using a non-parametric Wilcoxon tests in JMP® 12.12 software. The weighted and unweighted UniFrac distances for NPS and BAL samples were also compared using nonparametric ANOSIM test (analysis of similarities) with 999 Monte Carlo permutations.

To further explore the possible relationships between upper and lower respiratory tract microbial communities, multivariate analysis (linear correlation matrixes) was performed using JMP® 12.12 (SAS Institute Inc., North Carolina, USA). In view of the large number of comparisons, an additional, more conservative approach was adopted using multiple linear forward regression analysis. Forward linear regression analysis was performed using the SPSS version 22 (IBM Corp, Version 22.0, Armonk, NY, 2013) statistical package. Differences with a *P* value ≤0.05 were considered significant for all analyses.

Fastq data obtained in the current study were uploaded to the sequence read archive (SRA) on the National Center for Biotechnology Information (NCBI) website (http://www.ncbi.nlm.nih.gov/sra), to make the files available for a public database with bio-project accession number PRJNA323521.

## Results

### Sample population

Eight, six to eight month old, clinically healthy feedlot cattle were enrolled into the study. There was no detectable evidence of clinical signs of BRD in any of the enrolled calves. The median (range) of rectal temperature (C°), heart rate (beat/min), respiratory rate (breaths/min) and Whisper lung scores were 39.27 (38.55–39.38), 94 (64–118), 36 (26–48) and 1 (1–2) respectively.

### Overall sequence analysis

The sequencing analysis of the V3-V4 hypervariable regions of the bacterial 16S rRNA resulted in a total of 298,875 sequences in all NPS and BAL samples. The number of sequences per sample ranged from 2419.0 to 48,092.0 (mean 18,679.68, SD 13963.19) and comprised 195 OTUs (97% identity cutoff) across all samples (Additional file 1: Table S1).

### Taxonomic characterization of the nasopharyngeal versus bronchoalveolar microbiota

**Fig. 1:**
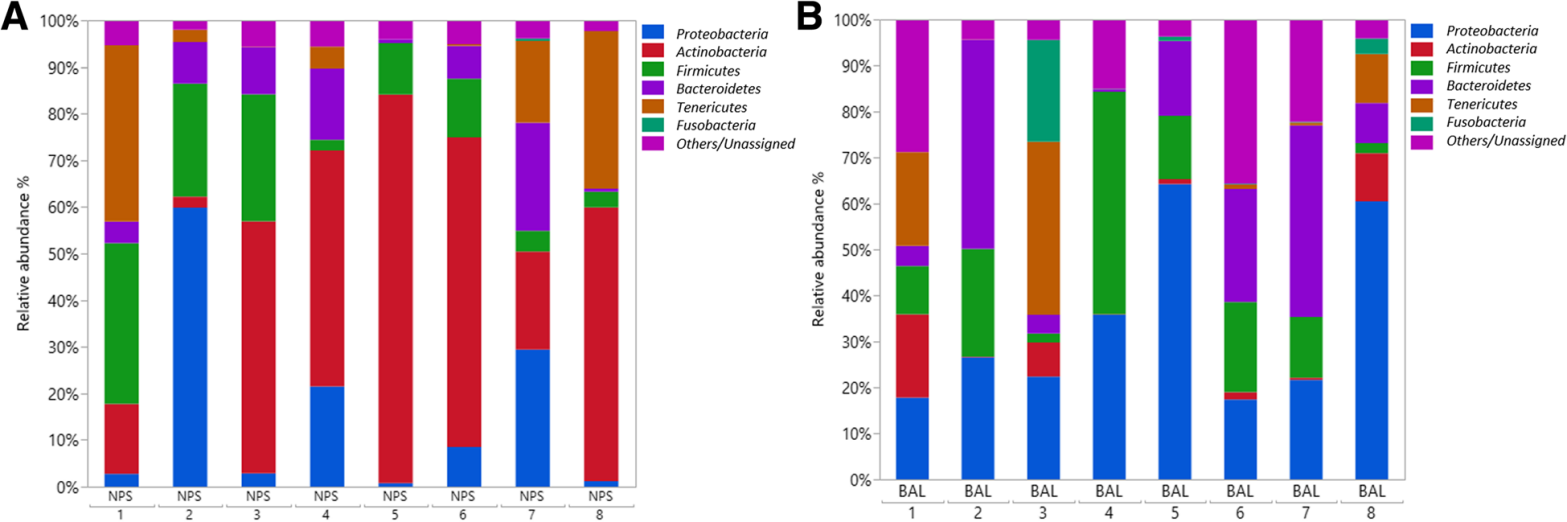
Relative abundance of bacterial 16S rRNA gene sequences at the phylum level observed in the NPS **a** and BAL **b** samples from 8 healthy feedlot calves. Only those bacterial phyla that averaged more than 1% of the relative abundance across all samples when sequencing V3-V4 hypervariable regions are displayed. All other unassigned and classified OTUs belonged to phyla comprising less than 1% of the total abundance represented as others/Unassigned

At the phylum level, the dominant bacterial phyla across all NPS and BAL samples were *Proteobacteria* (24.6%), *Actinobacteria* (24.4%), *Firmicutes* (15.8%), *Bacteroidetes* (13.5%) and *Tenericutes* (10.4%). Overall, 4.2% of NPS and 14.6% of BAL sequences could not be classified at the phylum-level (Fig. 1). The relative abundance of each phylum between individuals was highly variable across all the NPS and BAL samples (Fig. 1).

In NPS samples, we observed a predominance of *Actinobacteria* (43.9% versus 4.9% in BAL samples) and *Tenericutes* (12.1% versus 8.8% in BAL samples). While in BAL samples we observed a predominance of *Proteobacteria* (33.3% versus 15.9% in NPS samples), *Bacteroidetes* (18.2% versus 8.8% in NPS samples) and *Fusobacteria* (3.4% versus 0.1% in NPS samples).

**Fig. 2:**
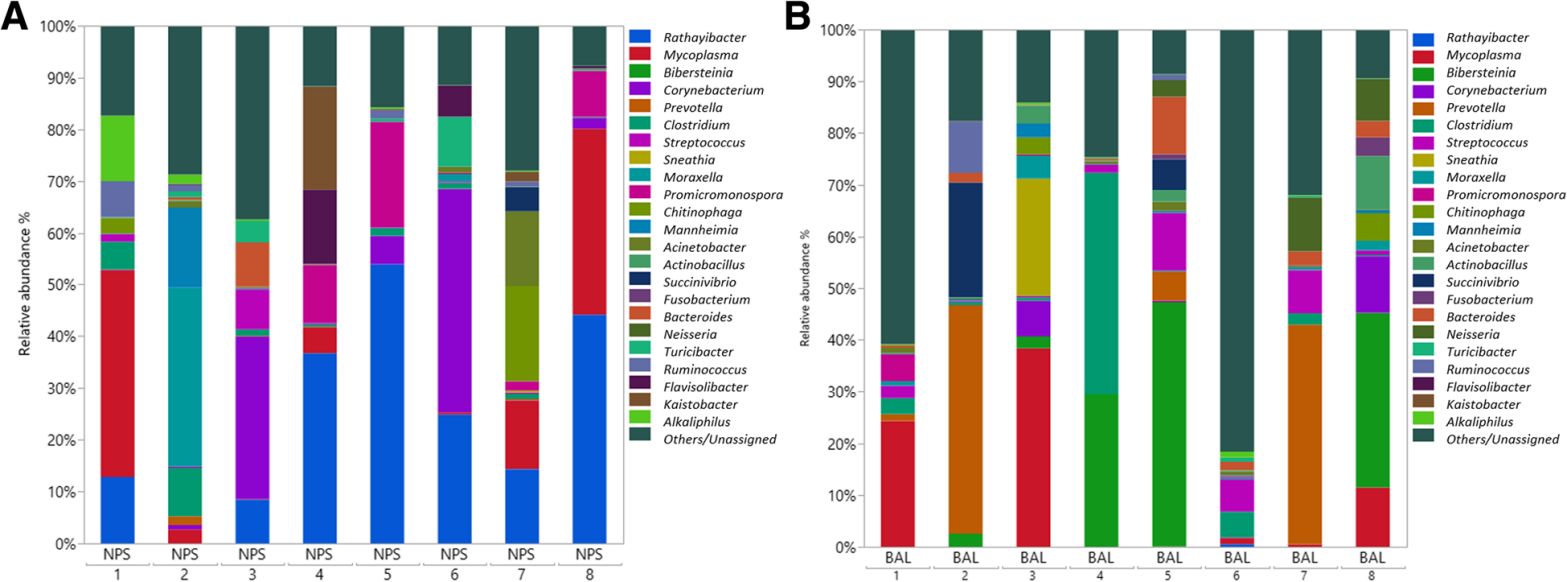
Relative abundance of bacterial 16S rRNA gene sequences at the genus level observed in the NPS **a** and BAL **b** samples from 8 healthy feedlot calves. Only those bacterial genera that averaged more than 1% of the relative abundance across all samples when sequencing V3-V4 hypervariable regions are displayed. All other unassigned and classified OTUs belonged to genera comprising less than 1% of the total abundance represented as others/Unassigned

At the genus level, there were 57 genus comprising greater than 0.1% of sequences in both NPS and BAL samples (Additional file 2: Table S2). The most abundant genera in the airway microbiota across all the NPS and BAL samples were *Rathayibacter* (12.24%), followed by *Mycoplasma* (10.84%), *Bibersteinia* (7.20%), *Corynebacterium* (6.32%), *Prevotella* (5.98%), and *Clostridium* (4.69%) (Fig. 2). There was a high inter-individual variability in the composition of the NPS and BAL microbiota across all the individuals (Fig. 2).

In NPS samples, we observed a predominance of *Rathayibacter* (24.41% versus 0.07% in BAL samples), *Mycoplasma* (12.20% versus 9.48% in BAL samples), and *Corynebacterium* (10.37% versus 2.28% in in BAL samples). While in BAL samples, we observed a predominance of *Bibersteinia* (14.41% versus 0% in NPS samples), *Prevotella* (11.69% versus 0.28% in NPS samples) and *Clostridium* (6.85% versus 2.53% in NPS samples). All other unassigned and classified OTUs belonged to genera comprising less than 1% of the total abundance represented as others/unassigned (Fig. 2).

Two bacterial phyla (*Proteobacteria* and *Actinobacteria*) showed a significant difference in relative abundance between NPS and BAL (*P* = 0.041 and 0.003 respectively). At the genus level, there were also statistical differences in abundances between these locations. For instance; *Rathayibacter* and *Flavisolibacter Micrococcus, Agrococcus* and *Cellulomonas* were more abundant in the NPS (*P* = 0.004, 0.028, 0.040, 0.009 and 0.002 respectively) while *Bibersteinia, Streptococcus* and *Bacteroides w*ere more abundant in the BAL samples (*P* = 0.012, 0.042 and 0.023 respectively). Interestingly, there were no significant differences in the relative abundance of the organisms commonly associated with BRD (*Mycoplasma, Mannheimia and Pasteurella,*) between the NPS and BAL samples (*P* = 0.512, 0.071 and 0.061 respectively).

**Fig. 3:**
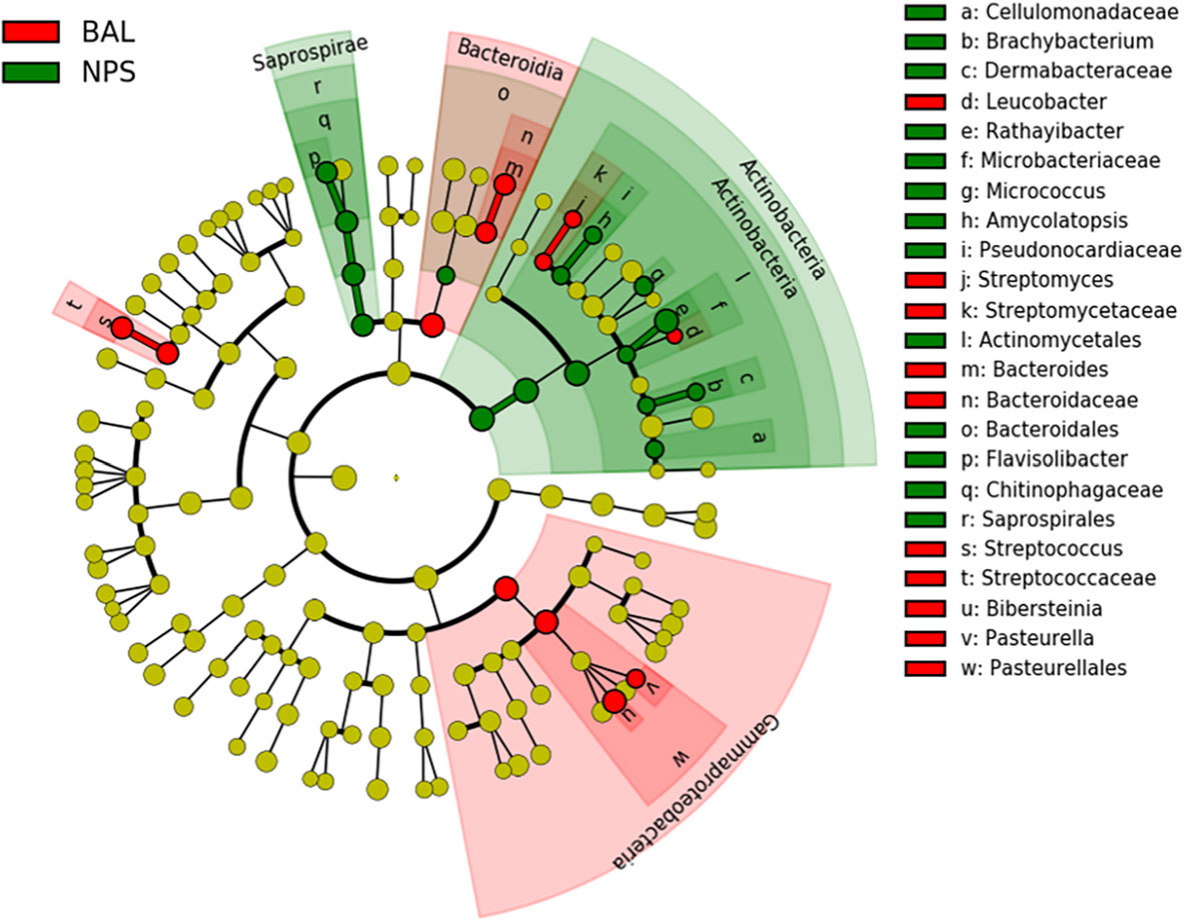
Cladogram displaying the 23 operational taxonomic units (OTUs) with significantly different abundance between the nasopharyngeal (NPS) and bronchoalveolar lavage (BAL) samples with an absolute Linear Discriminant Analysis LDA score log10 ≥ 2.0. Differences are represented in the color of the most abundant class (*red* indicating BAL, *Green* indicating NPS). Each circle’s diameter is proportional to the taxon’s relative abundance. Green: NPS; Red: BAL

**Fig. 4:**
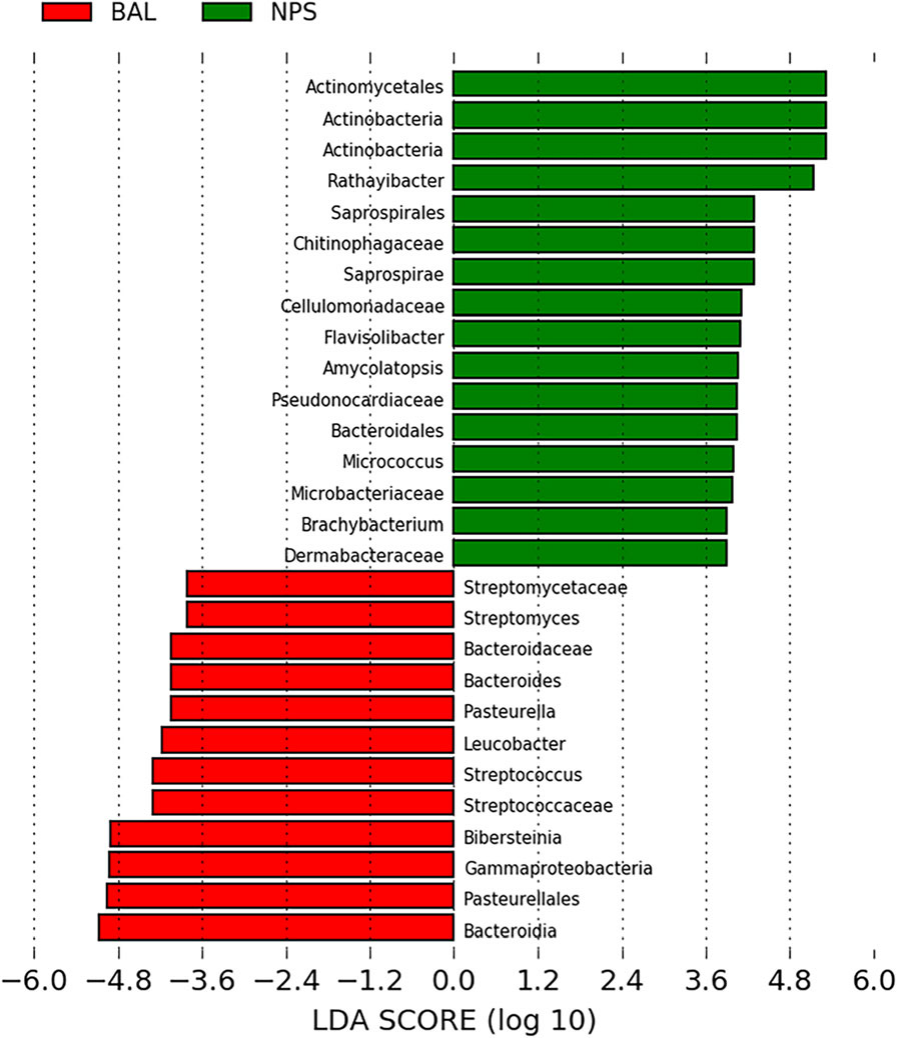
LEfSe comparison results of nasopharyngeal (NPS) and bronchoalveolar lavage (BAL) microbiota in clinically healthy calves depicting the top operational taxonomic units (OTUs) with the highest linear discriminant analysis LDA score log10 ≥ 2.0. These graphical outputs were generated by the publicly available LEfSe visualization modules. LEfSe scores can be interpreted as the degree of consistent difference in relative abundance between features in the two classes of analyzed microbial communities. Green: NPS; Red: BAL

LEfSe revealed that the NPS indicator OTUs were related to the bacterial genera *Brachybacterium, Rathayibacter, Micrococcus* and *Flavisolibacter.* Furthermore, the BAL indicator OTUs were related to the genera *Leucobacter, Bacteroides, Streptococcus, Bibersteinia* and *Pasteurella* (Fig. 3). The OTUs with the highest LDA (LDA log score threshold ≥2) from each group are depicted in (Fig. 4). The relative abundance of the selected microbial taxa that displayed significant differences between NPS and BAL are shown in (Additional file 3: Fig. S1).

### Relationship between upper and lower airway microbial community structure.

While there were both common and unique bacterial taxa present in both the NPS and BAL samples, the correlation analysis revealed strong associations between some of the most prevalent bacterial genera in both NPS (Additional file 4: Table S3 and Table S4) and BAL samples (Additional file 5: Table S5 and Table S6) at the population level, compatible with the concept of community structure.

There were highly significant correlations in the presence and abundance of some taxa (*Mycoplasma, Sneathia, Moraxella, Mannheimia, Succinivibrio, Ruminococcus* and *Flavisolibacter*) between the nasopharyngeal and lower respiratory samples (Table 1). In addition, the multiple linear forward regression analysis revealed that the presence of certain nasopharyngeal taxa was significantly associated with the presence of specific brochoalveolar taxa. For instance the presence of *Streptococcus* in NPS samples, was strongly predictive of the presence of *Moraxella and Mycoplasma* in BAL. Moreover, nasopharyngeal *Bacteroides, Promicromonospora* and *Mycoplasma* were strongly associated with the presence of brochoalveolar *Mannheimia, Bibersteinia* and *Mycoplasma* respectively (Table 2).

**Table 1:** Correlation between the most prevalent bacterial genera (those that averaged more than 1% of the relative abundance across all samples) between the nasopharyngeal (NPS) and bronchoalveolar lavage (BAL) samples in clinically healthy feedlot calves

NP/BAL bacterial genera	*Rathayibacter*	*Mycoplasma*	*Bibersteinia*	*Sneathia*	*Moraxella*	*Mannheimia*	*Succinivibrio*	*Ruminococcus*	*Flavisolibacter*
*Rathayibacter*			0.913**						
*Corynebacterium*	0.777*								
*Prevotella*							0.946**	0.983**	0.937**
*Clostridium*							0.826**	0.853**	0.771*
*Streptococcus*		0.877**		0.985**	0.917**	0.961**			
*Moraxella*							0.961**	0.993**	0.956**
*Promicromonospora*			0.965**						
*Mannheimia*							0.964**	0.995**	0.956**
*Fusobacterium*				0.879**	0.863**	0.917**			
*Bacteroides*		0.792*		0.999**	0.912**	0.979**			
*Turicibacter*	0.911**								

^*^ *P* value <0.05 ^**^ *P* value <0.01

The table shows the significant Pearson correlation coefficient between locations. Statistical significance is indicated with asterisks

**Table 2:** Multiple linear forward regression analysis showing inter-relationship between the specific nasopharyngeal (NPS) and bronchoalveolar (BAL) taxa in clinically healthy feedlot calves

NPS/BAL bacterial genera	*Bibersteinia* (BAL)			*Mycoplasma* (BAL)			*Moraxella* (BAL)			*Mannheimia* (BAL)		
	R square	SE of the estimate	*P* value	R Square	SE of the estimate	*P* value	R square	SE of the estimate	*P* value	R square	SE of the estimate	*P* value
*Mycoplasma* (NPS)				0.977	0.075	0.001						
*Streptococcus* (NPS)				0.769	0.025	0.004	0.842	0.006	0.001			
*Promicromonospora* (NPS)	0.913	0.054	0.0001									
*Actinobacillus* (NPS)	0.981	0.031	0.015									
*Bacteroides* (NPS)										0.950	0.002	0.0001

*SE* standard error

This analysis was focussed on the taxa commonly associated with bovine respiratory pathogens

### Overall variation in airway microbial community structure and diversity

The alpha diversity of the NPS and BAL communities in clinically healthy calves, was assessed using several indices (Table 3 and Additional file 6: Fig. S2). None of the alpha diversity indices differed significantly between NPS and BAL samples (*P* > 0.05).

**Table 3:** Bacterial diversity indices (Chao1, PD whole tree and Observed species) measures for the nasopharyngeal (NPS) and bronchoalveolar lavage (BAL) samples in clinically healthy feedlot calves

Calves group	Chao1 index	PD whole tree	Observed species
NPS	32.07 ± 9.35	2.77 ± 1.41	29.65 ± 8.32
BAL	28.75 ± 10.14	2.52 ± 1.03	24.22 ± 9.36

The data are presented as the mean ± standard deviation. There was no statistically significant difference in different bacterial diversity indices between the upper and lower respiratory tract *P* value >0.05

**Fig. 5:**
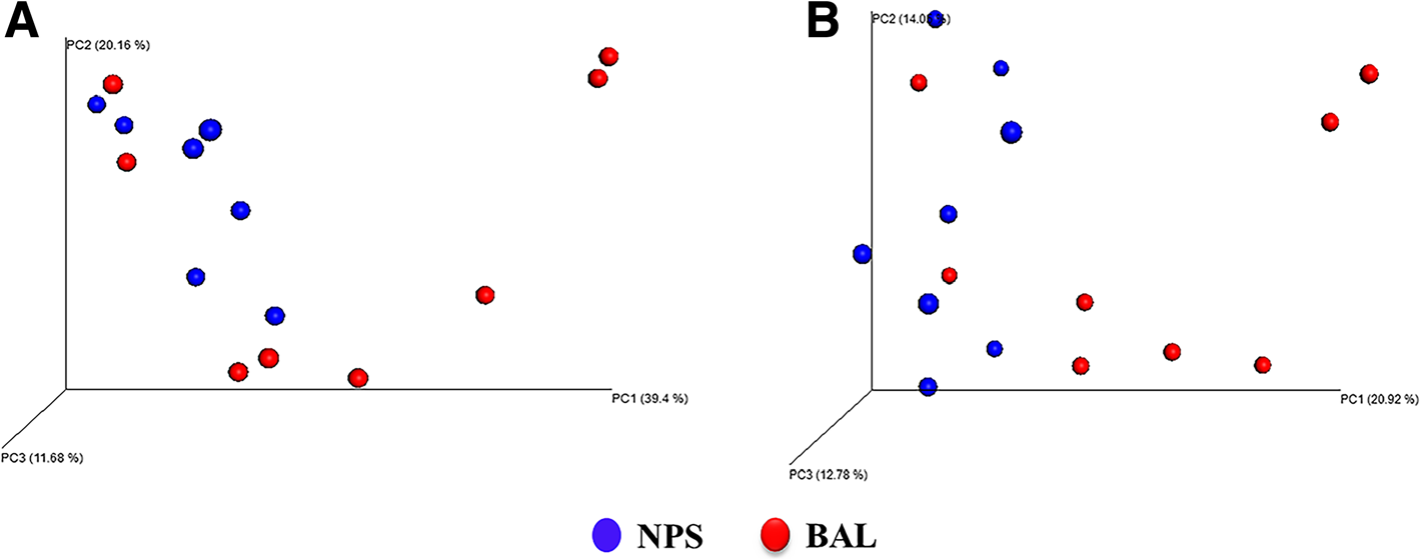
Principal coordinate analysis (PCoA) of the weighted UniFrac distances **a** and un-weighted UniFrac distances **b** for the nasopharyngeal (NPS) and bronchoalveolar lavage (BAL) samples in clinically healthy feedlot calves. The percent variation explained by each principal coordinate is indicated on the axes. The individual data points from NPS (*blue circle*) and BAL (*red circle*) which represent total airway microbiota compositions of each calf are also depicted

The microbial community structure (beta diversity) of the NPS and BAL samples were significantly different from one another (ANOSIM *R*-value = 0.448, *P* = 0.012, weighted Unifrac and *R*-value = 0.386, *P* = 0.022, unweighted Unifrac), indicating a clear distinction between the NPS and BAL communities (Fig. 5).

The core microbiota, defined as those OTUs found in all samples and identified at the genus level, of the respiratory tract was determined across all the NPS and BAL samples. In this population of animals, the core microbiota was composed of organisms from the *Mycoplasma, Clostridium, Streptococcus, Moraxella* and *Alkaliphilus* taxa.

## Discussion

In this study we describe the first use of high throughput sequencing in comparing the upper and lower respiratory microbiota of feedlot cattle. These findings are important because of the central role played by the upper respiratory microbial communities in defense against potential pathogen access and colonization of the lung and lower airways [9, 11] and because the terminal airways and adjoining alveoli are the primary site of pathogen colonization and pathology in respiratory infections.

The calves in this study all originated from the same cow-calf enterprise, and had been exposed to similar management and environmental challenges, and so the pattern of nasopharyngeal microbial communities was anticipated to be relatively consistent across individuals. Despite the uniform management conditions, there was a high inter-individual variability in the composition of the NPS and BAL microbiota at both the phylum (Fig. 1) and genus level (Fig. 2). This is compatible with broader studies that show the multifactorial determinants (genetic, epigenetic, environmental, age, sex and dietary) underlying the establishment of the mucosal microbiota [31]. While the nutritional and environmental exposures would have been similar between these animals in this study, there would have been a significant variability in genetic and epigenetic influences across the population, which could have accounted for the observed differences.

In our feedlot population the most predominant phylum (*Proteobacteria)* and genera (*Rathayibacter, Mycoplasma, Bibersteinia, Corynebacterium, Prevotella,* and *Clostridium)* were found across all NPS and BAL samples, and contributed to a common core microbiota of *Mycoplasma, Clostridium, Streptococcus, Moraxella* and *Alkaliphilus* for both of these sites*.* While the composition of the nasopharyngeal communities had some overlap with those previously reported in cattle at entry or after 60 days in the feedlot [10] there were some striking differences in our animals, specifically the complete absence of *Carnobacterium* and *Shewanella*, and the relatively low abundance of *Mannheimia.*

The relatively high abundance of *Rathayibacter* and *Corynebacterium* genus in NPS samples in our study is probably related to the high abundance of these organisms in the feedlot environment [32, 33], and the fact that the nasal cavity is the first respiratory compartment exposed to microbes from the air, feed and water [10]. *Corynebacterium* species are also widely recognized as a common commensal of the skin and mucosal surfaces [34].

The relatively high abundance of the *Mycoplasma* genus in all NPS and BAL samples in our study, supports the observations of other investigators that members of this taxa (e.g. *Mycoplasma dispar* and *Mycoplasma bovirhinis)* are common, non-pathogenic inhabitants, and essential components of the core microbiota of the respiratory tract airways in healthy cattle [9, 34]. The high frequency of occurrence of *Mycoplasma* species in our study also highlights the advantage of using culture-independent techniques for the identification of organisms with fastidious growth requirements [10, 35].

While there was some overlap between the upper and lower respiratory tract microbial communities, there was strong evidence for a distinct bronchoalveolar microbiota. For instance, the *Bibersteinia* were identified in BAL samples but were absent from the nasopharynx. The *Bibersteinia* genus belongs to the family *Pasteurellaceae.* These organisms have been reported as commensals of the nasopharynx and tonsils of healthy domestic sheep [36] and have been implicated as potential pathogens causing pneumonia in big horn sheep [37]. *Bibersteinia* has also been identified in both healthy and diseased cattle [38], but its occurrence in bovid species is unpredictable [39]. Since this is the first time that *Bibersteinia* has been reported as a common occupant of the lower respiratory tract of feedlot calves, further studies are needed to help understand its potential role in host health and productivity.

A particularly interesting aspect of our study was the identification of strong correlations between the presence of several specific taxa in nasopharyngeal samples and those in the lower respiratory tract. While the number of animals in this study was relatively small, and from a single source, these correlations are compatible with the notion of a mutualistic inter-relationship between the microbial communities at these two biogeographical locations. It has been shown that the healthy human lung does not harbor consistent and distinct microbial communities, but instead possesses a variable microbiota that is highly correlated, and largely indistinguishable from that of the upper respiratory tract [4]. The situation is altered somewhat in smokers, where the lung microbiome differs from the nasopharynx, but resembles that of the mouth [16]. This raises interesting questions regarding the process and route of microbial migration or translocation to the lungs from the environment, upper respiratory or gastrointestinal tract. In our study, the microbial populations of the upper and lower respiratory tract were clearly different from one another, which provided assurance that the sampling technique was relatively effective in preventing significant cross contamination between the upper and lower respiratory airway. In addition, it was clear from the strong statistical correlations that, at each of the sites examined, the presence and abundance of one taxa was often closely related to that of another. In combination these findings support the idea that the microbes identified at each biogeographical location were not present in random haphazard patterns, but were inter-related and part of relatively structured populations. While this is the first report of the existence of a mutually interdependent, location-dependent microbiota community-structure along the respiratory tract of feedlot cattle, the concept aligns well with that currently accepted in human medicine [16, 40].

While the results of this study were compelling, and could open new avenues of investigation in bovine respiratory health, the three experimental limitations of our study somewhat hinder the applicability of these findings to the broader feedlot cattle population. Firstly, the analyses were performed on a relatively small number of animals. While this sample size was similar to that used in equivalent, published metagenomic studies in healthy and BRD-treated calves [2, 10], data from a larger cohort of animals is required before definitive conclusions can be made regarding the specific taxa that constitute the lower respiratory microbiota in feedlot populations. Secondly, the potential for bronchoscopic contamination, or carryover, from the upper airway during passage of the external tube, could be viewed as potentially skewing the BAL microbial populations towards that present in the oropharynx or nasopharynx. The BAL technique was selected over a trans-tracheal approach, because we were interested in harvesting secretions from the smaller, more distal, airways as representative of the lower lung/airway microbiota. While we consider that the nasal approach circumvented the risk of significant oropharyngeal contamination, it was interesting to note that the NPS and BAL microbial populations in our study were clearly distinct from one another. It is possible that the high volume of fluid used for the BAL sampling procedure diluted the impact of any minor nasopharyngeal contamination on our results and conclusions. The last potential drawback in the study design was the absence of inclusion of negative controls in the sample analysis. It has been recently shown that a control sample, at the level of DNA extraction and processing, can mitigate the effects of contamination when examining low biomass environments [41]. At the time of this study, the inclusion of negative sequencing controls was not a widespread practice, and was not a part of equivalent studies [7, 9]. In order to overcome the potential impact of low abundance contaminants on our results, a conservative approach to data interpretation was adopted. This approach meant that our analysis, interpretation, discussion, and conclusions were focused entirely on high abundance taxa, and on those populations that were significantly different between the two biogeographical locations.

As additional studies examining the respiratory microbiota are conducted in larger cohorts of animals from different environments, the notion of a relationship between the upper and lower respiratory tract microbiota could be a catalyst for a new trajectory of investigation in to bovine respiratory health. This is particularly exciting in view if the importance of BRD in the health, welfare and productivity of the cattle industry, and with regard to the potential for developing strategies that could help reduce the use of antimicrobials in this important branch of livestock-based food production.

## Conclusion

In conclusion, this is the first study to describe the relationship between NPS and BAL microbiota in healthy feedlot cattle. In this cohort of cattle, the respiratory tract microbiota was dominated by *Proteobacteria* and *Actinobacteria.* The significant differences in the microbial community structure of the NPS and BAL samples indicated that a clear distinction exists between the microbiota at these sites. However, the strong correlations between the presence of several specific taxa in nasopharyngeal samples and those in the lower respiratory tract supports the notion of the existence of a mutualistic inter-relationship between these biogeographically disparate microbial communities. Future studies, in large cohorts of animals, are needed to determine the role and clinical importance of the relationships of respiratory tract microbial communities with health, productivity, and susceptibility to the development of respiratory disease, in growing cattle.

## Additional files

Additional file 1: Table S1. OTUs found in all NPS and BAL samples from 8 healthy feedlot calves. (XLSX 28 kb)

Additional file 2: Table S2. Relative abundance of the most common bacterial taxa at genus level for the NPS and BAL samples in clinically healthy feedlot calves. For each group the mean relative of predominant bacterial genera is reported. We determined statistically significant differences in relative abundance between groups (NPS and BAL) using nonparametric Wilcoxon tests.; *P*-values were calculated when statistically significant (*P* < 0.05). NS: non-significant *P*-value. (DOCX 17 kb)

Additional file 3: Fig. S1. Selected microbial taxa displaying significant differences in relative abundance between NPS and BAL. X-axis represent the relative abundance and Y-axis represent the individual samples. Straight line represents the mean abundance value of the group and the Dotted Line represents the median of the group. (DOCX 776 kb)

Additional file 4: Table S3. Correlation between the most prevalent bacterial genera in the nasopharyngeal swab samples. **Table S4.** Multiple linear forward regression analysis showing inter-relationship between the specific nasopharyngeal taxa in clinically healthy feedlot calves. (DOCX 15 kb)

Additional file 5: Table S5. Correlation between the most prevalent bacterial genera in the bronchoalveolar lavage samples. **Table S6.** Multiple linear forward regression analysis showing inter-relationship between the specific bronchoalveolar taxa in clinically healthy feedlot calves. (DOCX 16 kb)

Additional file 6: Fig. S2. Rarefaction curves of 16S rRNA gene sequences obtained from nasopharyngeal samples (NPS) and bronchoalveolar lavage samples (BAL) form clinically healthy calves. The graphical lines represent the mean and error bars represent standard deviations. The analysis was performed on a randomly selected subset of 2400 sequences per sample. (DOCX 194 kb)

## Abbreviations

BAL: Broncho alveolar lavage
BRD: Bovine respiratory disease
DNA: Deoxyribonucleic acid
LDA: Linear discriminant analysis
LEfSe: Linear discriminant analysis effective size
NPS: Nasopharyngeal swabs
OTU: Operational taxonomic unit
PCoA: Principle coordinate analysis
QIIME: Quantitative Insights Into Microbial Ecology
rRNA: Ribosomal ribo nucleic acid
