# Supplementary material for: Relationship between nasopharyngeal and bronchoalveolar microbial communities in clinically healthy feedlot cattle
Source: BMC Microbiol. 2017 Jun 23;17:138. doi: 10.1186/s12866-017-1042-2 (PMC5481913; doi:10.1186/s12866-017-1042-2)
Supplement: Supplementary file 2 — Relative abundance of the most common bacterial taxa at genus level for the NPS and BAL samples in clinically healthy feedlot calves. For each group the mean relative of predominant bacterial genera is reported. We determined statistically significant differences in relative abundance between groups (NPS and BAL) using nonparametric Wilcoxon tests.; P-values were calculated when statistically significant (P < 0.05). NS: non-significant P-value. (DOCX 17 kb) [file 12866_2017_1042_MOESM2_ESM.docx]

**Table S2.** Relative abundance of the most common bacterial taxa at genus level for the NPS and BAL samples in clinically healthy feedlot calves. For each group the mean relative of predominant bacterial genera is reported. We determined statistically significant differences in relative abundance between groups (NPS and BAL) using nonparametric Wilcoxon tests.; *P*-values were calculated when statistically significant (P<0.05). NS: non-significant *P*-value.

| **Phylum** | **Genus** | **Overall relative abundance** | **BAL** | **NPS** | **P value** |
| --- | --- | --- | --- | --- | --- |
| *Actinobacteria* | *Rathayibacter* | 0.1224 | 0.001 ±0.002 | 0.244±0.189 | **0.004** |
| *Tenericutes* | *Mycoplasma* | 0.1085 | 0.095±0.145 | 0.122±0.165 | NS |
| *Proteobacteria* | *Bibersteinia* | 0.0721 | 0.144±0.192 | 0 | **0.012** |
| *Actinobacteria* | *Corynebacterium* | 0.0633 | 0.023±0.043 | 0.104±0.170 | NS |
| *Bacteroidetes* | *Prevotella* | 0.0599 | 0.117±0.196 | 0.003±0.006 | NS |
| *Firmicutes* | *Clostridium* | 0.0470 | 0.069±0.146 | 0.025±0.032 | NS |
| *Firmicutes* | *Streptococcus* | 0.0259 | 0.039±0.042 | 0.013±0.026 | **0.04** |
| *Fusobacteria* | *Sneathia* | 0.0144 | 0.028±0.080 | 0.0004±0.001 | NS |
| *Proteobacteria* | *Moraxella* | 0.0278 | 0.010±0.015 | 0.046±0.121 | NS |
| *Actinobacteria* | *Promicromonospora* | 0.0300 | 0.007±0.018 | 0.053±0.075 | NS |
| *Bacteroidetes* | *Chitinophaga* | 0.0187 | 0.011±0.021 | 0.027±0.064 | NS |
| *Proteobacteria* | *Mannheimia* | 0.0125 | 0.006±0.009 | 0.020±0.055 | NS |
| *Proteobacteria* | *Acinetobacter* | 0.0138 | 0.006±0.006 | 0.021±0.050 | NS |
| *Proteobacteria* | *Actinobacillus* | 0.0107 | 0.021±0.036 | 0.001±0.001 | NS |
| *Proteobacteria* | *Succinivibrio* | 0.0206 | 0.035±0.078 | 0.006±0.017 | NS |
| *Fusobacteria* | *Fusobacterium* | 0.0030 | 0.006±0.013 | 0.00004±0.00005 | NS |
| *Bacteroidetes* | *Bacteroides* | 0.0191 | 0.027±0.036 | 0.012±0.030 | **0.02** |
| *Proteobacteria* | *Neisseria* | 0.0136 | 0.027±0.042 | 0 | NS |
| *Firmicutes* | *Turicibacter* | 0.0107 | 0.002±0.003 | 0.020±0.034 | NS |
| *Firmicutes* | *Ruminococcus* | 0.0135 | 0.014±0.035 | 0.013±0.024 | NS |
| *Bacteroidetes* | *Flavisolibacter* | 0.0133 | 0.00002±0.0001 | 0.027±0.052 | **0.02** |
| *Proteobacteria* | *Kaistobacter* | 0.0136 | 0.0000±0.0000 | 0.027±0.070 | NS |
| *Firmicutes* | *Alkaliphilus* | 0.0111 | 0.003±0.004 | 0.019±0.044 | NS |
| *Bacteroidetes* | *Paludibacter* | 0.0165 | 0.0330±0.0910 | 0.00001±0.0009 | NS |
| *Actinobacteria* | *Oerskovia* | 0.0088 | 0 | 0.0176±0.0489 | NS |
| *Actinobacteria* | *Parascardovia* | 0.0087 | 0.0174±0.0493 | 0 | NS |
| *Tenericutes* | *Ureaplasma* | 0.0056 | 0.0006±0.0016 | 0.0106±0.0206 | NS |
| *Firmicutes* | *Peptococcus* | 0.0045 | 0.0001±0.0003 | 0.0088±0.0229 | NS |
| *Firmicutes* | *Blautia* | 0.0042 | 0.0041±0.0045 | 0.0043±0.0075 | NS |
| *Bacteroidetes* | *Wautersiella* | 0.0038 | 0 | 0.0076±0.0216 | NS |
| *Firmicutes* | *Lactobacillus* | 0.0033 | 0.0055±0.0083 | 0.0012±0.0009 | NS |
| *Firmicutes* | *Trichococcus* | 0.0033 | 0.0007±0.0009 | 0.0058±0.0075 | NS |
| *Proteobacteria* | *Salinivibrio* | 0.0032 | 0 | 0.0016±0.0046 | NS |
| *Firmicutes* | *Jeotgalicoccus* | 0.0031 | 0.0002±0.004 | 0.0061±0.0160 | NS |
| *Firmicutes* | *Sarcina* | 0.0031 | 0.0059±0.0145 | 0.0004±0.0009 | NS |
| *Proteobacteria* | *Escherichia* | 0.0031 | 0.0061±0.0133 | 0.00004±0.0001 | NS |
| *Firmicutes* | *Facklamia* | 0.0031 | 0.0001±0.0004 | 0.0060±0.0125 | NS |
| *Proteobacteria* | *Tolumonas* | 0.0030 | 0.0003±0.0008 | 0.0057±0.0161 | NS |
| *Proteobacteria* | *Gallibacterium* | 0.0028 | 0.0056±0.0102 | 0.0001±0.0001 | NS |
| *Proteobacteria* | *Devosia* | 0.0028 | 0.0004±0.0012 | 0.0052±0.0135 | NS |
| *Proteobacteria* | *Psychrobacter* | 0.0026 | 0.0007±0.0012 | 0.0045±0.0081 | NS |
| *Firmicutes* | *Megasphaera* | 0.0024 | 0.0047±0.0092 | 0.0001±0.0001 | NS |
| *Firmicutes* | *Enterococcus* | 0.0022 | 0.0001±0.0003 | 0.0042±0.0093 | NS |
| *Actinobacteria* | *Micrococcus* | 0.0022 | 0.00001±0.00004 | 0.0043±0.0086 | **0.04** |
| *Firmicutes* | *Butyrivibrio* | 0.0019 | 0.0037±0.0087 | 0.0001±0.0001 | NS |
| *Actinobacteria* | *Agrococcus* | 0.0019 | 0.0003±0.0008 | 0.0035±0.0041 | **0.009** |
| *Proteobacteria* | *Paracoccus* | 0.0017 | 0 | 0.0035±0.0097 | NS |
| *Firmicutes* | *Selenomonas* | 0.0016 | 0.0031±0.0087 | 0.000006±0.00001 | NS |
| *Proteobacteria* | *Pelomonas* | 0.0015 | 0.0005±0.0015 | 0.0025±0.0070 | NS |
| *Bacteroidetes* | *Dysgonomonas* | 0.0012 | 0.0012±0.0023 | 0.0012±0.0032 | NS |
| *Actinobacteria* | *Cellulomonas* | 0.0012 | 0.0001±0.0003 | 0.0023±0.0019 | **0.002** |
| *Firmicutes* | *Anaerofilum* | 0.0011 | 0 | 0.0022±0.0063 | NS |
| *Proteobacteria* | *Pasteurella* | 0.0011 | 0.0020±0.0022 | 0.0002±0.0002 | NS |
| *Proteobacteria* | *Pseudomonas* | 0.0011 | 0.0016±0.0025 | 0.0005±0.0009 | NS |
| *Firmicutes* | *Negativicoccus* | 0.0011 | 0.0021±0.0060 | 0.00002±0.0001 | NS |
| *Proteobacteria* | *Campylobacter* | 0.0010 | 0.0018±0.0036 | 0.0002±0.0002 | NS |
| *Firmicutes* | *Salinicoccus* | 0.0010 | 0 | 0.0020±0.0056 | NS |
