# Supplementary material for: Relationship between nasopharyngeal and bronchoalveolar microbial communities in clinically healthy feedlot cattle
Source: BMC Microbiol. 2017 Jun 23;17:138. doi: 10.1186/s12866-017-1042-2 (PMC5481913; doi:10.1186/s12866-017-1042-2)
Supplement: Supplementary file 3 — Selected microbial taxa displaying significant differences in relative abundance between NPS and BAL. X-axis represent the relative abundance and Y-axis represent the individual samples. Straight line represents the mean abundance value of the group and the Dotted Line represents the median of the group. (DOCX 776 kb) [file 12866_2017_1042_MOESM3_ESM.docx]

**Figure S1.** Selected microbial taxa displaying significant differences in relative abundance between NPS and BAL. X-axis represent the relative abundance and Y-axis represent the individual samples. Straight line represents the mean abundance value of the group and the Dotted Line represents the median of the group.

**
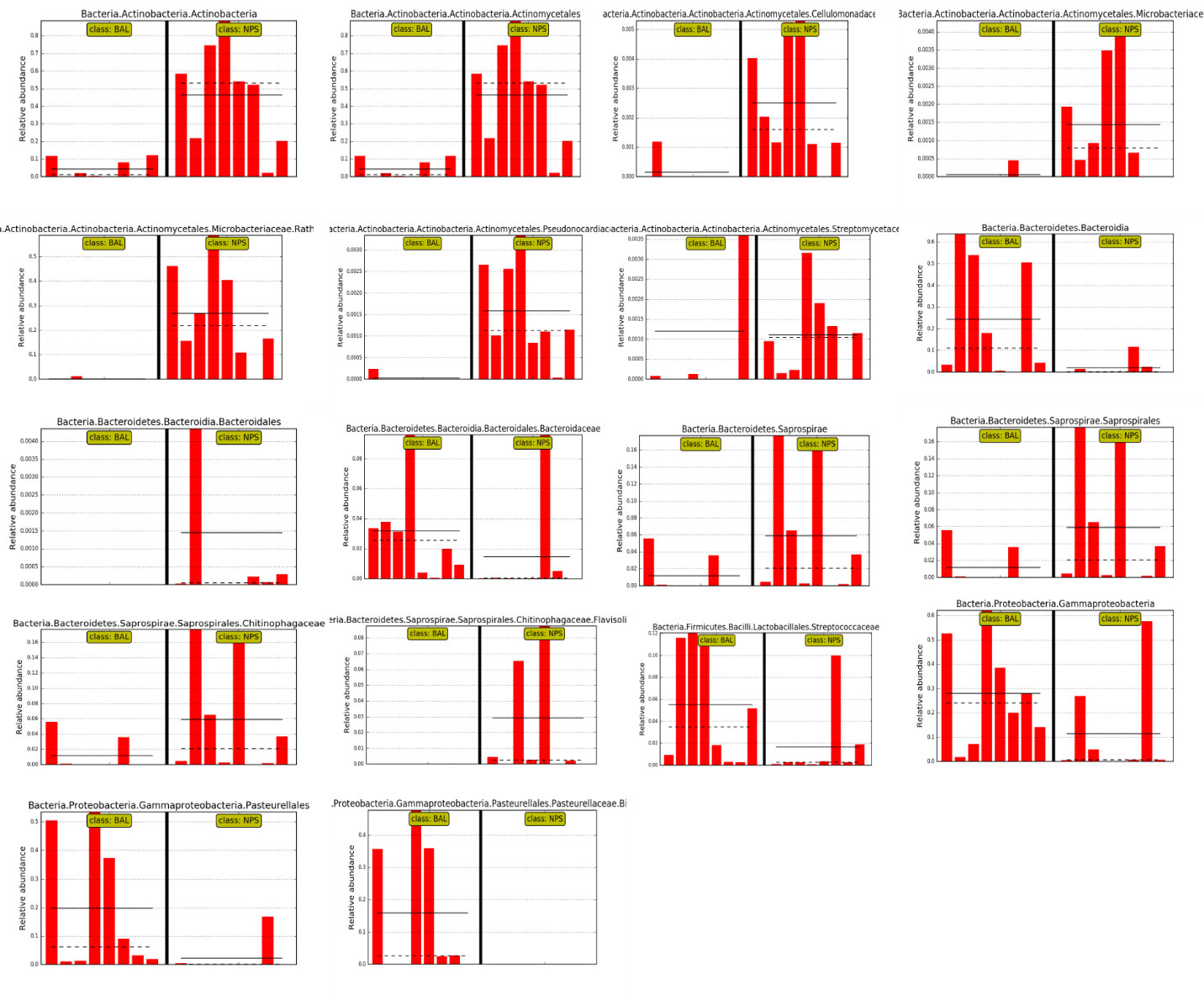
**
