# Supplementary material for: Relationship between nasopharyngeal and bronchoalveolar microbial communities in clinically healthy feedlot cattle
Source: BMC Microbiol. 2017 Jun 23;17:138. doi: 10.1186/s12866-017-1042-2 (PMC5481913; doi:10.1186/s12866-017-1042-2)
Supplement: Supplementary file 4 — Correlation between the most prevalent bacterial genera in the nasopharyngeal swab samples. Table S4. Multiple linear forward regression analysis showing inter-relationship between the specific nasopharyngeal taxa in clinically healthy feedlot calves. (DOCX 15 kb) [file 12866_2017_1042_MOESM4_ESM.docx]

**Table S3.** Correlation between the most prevalent bacterial genera in the nasopharyngeal swab samples.

| NPS  bacterial genera | ***Prevotella*** | ***Clostridium*** | ***Streptococcus*** | ***Sneathia*** | ***Moraxella*** | ***Chitinophaga*** | ***Mannheimia*** | ***Acinetobacter*** | ***Succinivibrio*** | ***Fusobacterium*** | ***Bacteroides*** |
| --- | --- | --- | --- | --- | --- | --- | --- | --- | --- | --- | --- |
| ***Prevotella*** | 1** | 0.839** |  |  | 0.991** |  | 0.991** |  |  |  |  |
| ***Clostridium*** | 0.839** | 1** |  |  | 0.857** |  | 0.858** |  |  |  |  |
| ***Streptococcus*** |  |  | 1** |  |  |  |  |  |  | 0.831** | 0.982** |
| ***Sneathia*** |  |  |  | 1** |  | 0.988** |  | 0.995** | 0.999** |  |  |
| ***Moraxella*** | 0.991** | 0.856** |  |  | 1** |  | 0.999** |  |  |  |  |
| ***Chitinophaga*** |  |  |  | 0.988** |  | 1** |  | 0.979** | 0.989** |  |  |
| ***Mannheimia*** | 0.991** | 0.858** |  |  | 0.999** |  | 1** |  |  |  |  |
| ***Acinetobacter*** |  |  |  | 0.995** |  | 0.979** |  | 1** | 0.994** |  |  |
| ***Actinobacillus*** |  |  |  |  |  |  |  |  |  |  |  |
| ***Succinivibrio*** |  |  |  | 0.999** |  | 0.989** |  | 0.994** | 1** |  |  |
| ***Fusobacterium*** |  |  | 0.831** |  |  |  |  |  |  | 1** | 0.891** |
| ***Bacteroides*** |  |  | 0.982** |  |  |  |  |  |  | 0.891** | 1** |

** p value < 0.01

**Table S4.** Multiple linear forward regression analysis showing inter-relationship between the specific nasopharyngeal taxa in clinically healthy feedlot calves.

| NPS  bacterial genera | ***Prevotella*** | | | ***Clostridium*** | | | ***Moraxella*** | | | ***Mannheimia*** | | |
| --- | --- | --- | --- | --- | --- | --- | --- | --- | --- | --- | --- | --- |
|  | R  square | SE of the estimate | P  value | R  square | SE of the estimate | P value | R  square | SE of the estimate | P value | R  square | SE of the estimate | P value |
| ***Mannheimia*** | 0.983 | 0.008 | 0.0001 | 0.693 | 0.017 | 0.006 | 0.999 | 0.005 | 0.0001 |  |  |  |
| ***Moraxella*** |  |  |  |  |  |  |  |  |  | 0.999 | 0.002 | 0.0001 |
| ***Acintobacter*** | 0.994 | 0.005 | 0.024 |  |  |  |  |  |  |  |  |  |
| ***Fusobacterium*** |  |  |  |  |  |  | 0.998 | 0.002 | 0.0001 | 0.998 | 0.001 | 0.0001 |
| ***Mycoplasma*** |  |  |  |  |  |  |  |  |  |  |  |  |
| ***Turicibacter*** |  |  |  |  |  |  | 0.998 | 0.002 | 0.001 | 0.998 | 0.007 | 0.001 |
| ***Ruminococcus*** |  |  |  | 0.984 | 0.004 | 0.0001 |  |  |  |  |  |  |
| ***Corynbacterium*** |  |  |  | 0.997 | 0.002 | 0.013 |  |  |  |  |  |  |
| ***Bacteroides*** | 0.999 | 0.001 | 0.004 |  |  |  |  |  |  |  |  |  |

SE standard error
