# Supplementary material for: Relationship between nasopharyngeal and bronchoalveolar microbial communities in clinically healthy feedlot cattle
Source: BMC Microbiol. 2017 Jun 23;17:138. doi: 10.1186/s12866-017-1042-2 (PMC5481913; doi:10.1186/s12866-017-1042-2)
Supplement: Supplementary file 5 — Correlation between the most prevalent bacterial genera in the bronchoalveolar lavage samples. Table S6. Multiple linear forward regression analysis showing inter-relationship between the specific bronchoalveolar taxa in clinically healthy feedlot calves. (DOCX 16 kb) [file 12866_2017_1042_MOESM5_ESM.docx]

**Table S5.** Correlation between the most prevalent bacterial genera in the bronchoalveolar lavage samples.

| **BAL**  **bacterial genera** | ***Rathayibacter*** | ***Mycoplasma*** | ***Corynebacterium*** | ***Sneathia*** | ***Moraxella*** | ***Chitinophaga*** | ***Mannheimia*** | ***Actinobacillus*** | ***Succinivibrio*** | ***Fusobacterium*** | ***Turicibacter*** | ***Ruminococcus*** | ***Flavisolibacter*** | ***Alkaliphilus*** |
| --- | --- | --- | --- | --- | --- | --- | --- | --- | --- | --- | --- | --- | --- | --- |
| ***Rathayibacter*** | 1** |  |  |  |  |  |  |  |  |  | 0.901** |  |  | 0.935** |
| ***Mycoplasma*** |  | 1** |  | 0.803** | 0.893** |  | 0.82** |  |  |  |  |  |  |  |
| ***Corynebacterium*** |  |  | 1** |  | 0.72* | 0.999** |  | 0.939** |  | 0.778* |  |  |  |  |
| ***Sneathia*** |  | 0.803** |  | 1** | 0.919** |  | 0.982** |  |  |  |  |  |  |  |
| ***Moraxella*** |  | 0.893** | 0.72* | 0.919** | 1** | 0.723* | 0.955** |  |  |  |  |  |  |  |
| ***Chitinophaga*** |  |  | 0.999** |  | 0.723* | 1** |  | 0.934** |  | 0.771* |  |  |  |  |
| ***Mannheimia*** |  | 0.82** |  | 0.982** | 0.955** |  | 1** |  |  |  |  |  |  |  |
| ***Actinobacillus*** |  |  | 0.939** |  |  | 0.934** |  | 1** |  | 0.942** |  |  |  |  |
| ***Succinivibrio*** |  |  |  |  |  |  |  |  | 1** |  |  | 0.986** | 0.912** |  |
| ***Fusobacterium*** |  |  | 0.778* |  |  | 0.771* |  | 0.942** |  | 1** |  |  |  |  |
| ***Turicibacter*** | 0.906** |  |  |  |  |  |  |  |  |  | 1** |  |  | 0.819** |
| ***Ruminococcus*** |  |  |  |  |  |  |  |  | 0.986** |  |  | 1** | 0.949** |  |
| ***Flavisolibacter*** |  |  |  |  |  |  |  |  | 0.912** |  |  | 0.949** | 1** |  |
| ***Alkaliphilus*** | 0.935** |  |  |  |  |  |  |  |  |  | 0.819** |  |  | 1** |

* p value < 0.05. ** p value < 0.01

**Table S6.** Multiple linear forward regression analysis showing inter-relationship between the specific bronchoalveolar taxa in clinically healthy feedlot calves.

| BAL  Bacterial genera | ***Corynebacterium*** | | | ***Mycoplasma*** | | | ***Moraxella*** | | | ***Mannheimia*** | | |
| --- | --- | --- | --- | --- | --- | --- | --- | --- | --- | --- | --- | --- |
|  | R  square | SE of the estimate | P  value | R  square | SE of the estimate | P value | R  square | SE of the estimate | P value | R  square | SE of the estimate | P value |
| ***Moraxella*** |  |  |  | 0.797 | 0.07 | 0.003 |  |  |  |  |  |  |
| ***Mannheimia*** |  |  |  | 0.999 | 0.005 | 0.0001 | 0.912 | 0.005 | 0.0001 |  |  |  |
| ***Sneathia*** |  |  |  |  |  |  |  |  |  | 0.964 | 0.002 | 0.0001 |
| ***Fusobacterium*** |  |  |  |  |  |  |  |  |  | 0.988 | 0.001 | 0.0001 |
| ***Promicromonospora*** |  |  |  | 0.992 | 0.015 | 0.0001 |  |  |  |  |  |  |
| ***Acinetobacter*** | 0.999 | 0.008 | 0.0001 |  |  |  |  |  |  |  |  |  |
| ***Chitinophaga*** | 0.998 | 0.001 | 0.0001 |  |  |  |  |  |  |  |  |  |

SE standard error
