# Supplementary material for: Relationship between nasopharyngeal and bronchoalveolar microbial communities in clinically healthy feedlot cattle
Source: BMC Microbiol. 2017 Jun 23;17:138. doi: 10.1186/s12866-017-1042-2 (PMC5481913; doi:10.1186/s12866-017-1042-2)

**Figure S2:** Rarefaction curves of 16S rRNA gene sequences obtained from nasopharyngeal samples (NPS) and bronchoalveolar lavage samples (BAL) form clinically healthy calves. The graphical lines represent the mean and error bars represent standard deviations. The analysis was performed on a randomly selected subset of 2400 sequences per sample.


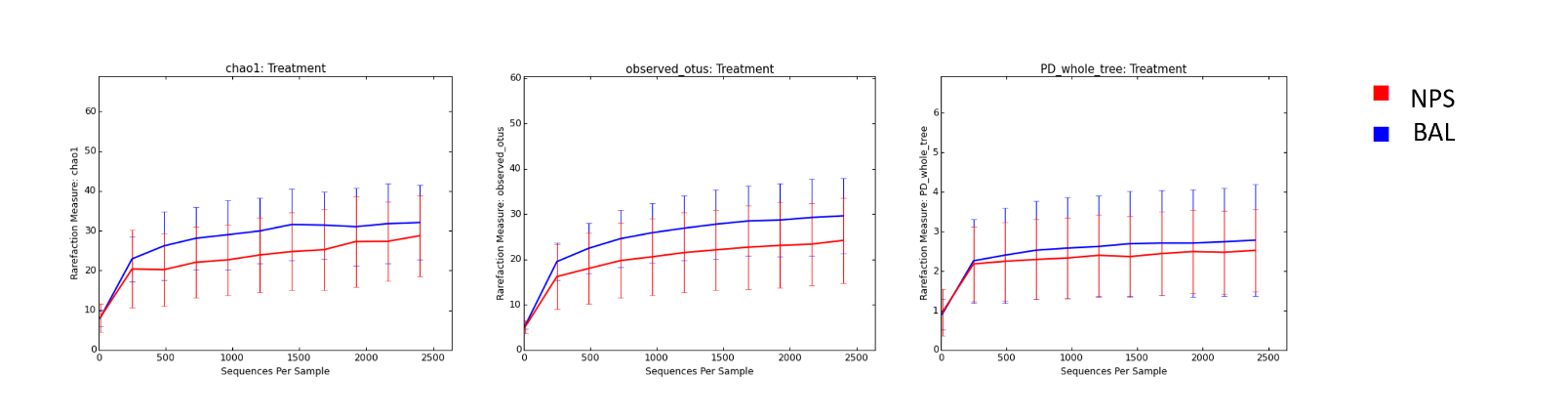

Supplement: Supplementary file 6 — Rarefaction curves of 16S rRNA gene sequences obtained from nasopharyngeal samples (NPS) and bronchoalveolar lavage samples (BAL) form clinically healthy calves. The graphical lines represent the mean and error bars represent standard deviations. The analysis was performed on a randomly selected subset of 2400 sequences per sample. (DOCX 194 kb) [file 12866_2017_1042_MOESM6_ESM.docx]
